# Localised Ag^+^ vibrations at the origin of ultralow thermal conductivity in layered thermoelectric AgCrSe_2_

**DOI:** 10.1038/srep23415

**Published:** 2016-03-22

**Authors:** F. Damay, S. Petit, S. Rols, M. Braendlein, R. Daou, E. Elkaïm, F. Fauth, F. Gascoin, C. Martin, A. Maignan

**Affiliations:** 1 Laboratoire Léon Brillouin, CEA-CNRS UMR 12, 91191 GIF-SUR-YVETTE CEDEX, France; 2 Institut Laue-Langevin, 6 rue Jules Horowitz, BP 156, 38042 GRENOBLE CEDEX 9, France; 3 Laboratoire CRISMAT, CNRS UMR 6508, 6 bvd Maréchal Juin, 14050 CAEN CEDEX, France; 4 Synchrotron Soleil, Saint-Aubin BP 48, 91192 GIF-SUR-YVETTE CEDEX, France; 5 Synchrotron ALBA, Carretera BP 1413, 08290 Cerdanyola del Vallès, BARCELONA, Spain

## Abstract

In materials science, the substructure approach consists in imagining complex materials in which a particular property is associated with a distinct structural feature, so as to combine different chosen physical characteristics, which otherwise have little chance to coexist. Applied to thermoelectric materials, it has been used to achieve simultaneously phonon-glass and electron-crystal properties. Mostly studied for its superionic conductivity, AgCrSe_2_ is a naturally layered compound, which achieves very low thermal conductivity, ~0.4 W.K^−1^.m^−1^ at RT (room temperature), and is considered a promising thermoelectric. The Cr atoms of the [CrSe_2_]_∞_ layer bear a spin S = 3/2, which orders below T_N_ = 55 K. Here we report low temperature inelastic neutron scattering experiments on AgCrSe_2_, alongside the magnetic field evolution of its thermal and electrical transport. We observe a very low frequency mode at 3 meV, ascribed to large anharmonic displacements of the Ag^+^ ions in the [Ag]_∞_ layer, and 2D magnetic fluctuations up to 3 T_N_ in the chromium layer. The low thermal conductivity of AgCrSe_2_ is attributed to acoustic phonon scattering by a regular lattice of Ag^+^ oscillating in quasi-2D potential wells. These findings highlight a new way to achieve localised phonon modes in a perfectly crystalline solid.

AgCrSe_2_ has a relatively simple polar crystal structure at room temperature^1^,^2^, of the *R*3*m* layered type (*a* = *b* = 3.6836(2) Å and *c* = 21.2375(12) Å at 300 K), consisting of layers of edge-sharing CrSe_6_ octahedra separated by Ag^+^ ions in tetrahedral sites. The latter actually form a pseudo two-dimensional puckered honeycomb lattice made of two interpenetrating triangular sublattices α and β (Fig. 1a,b). Below the order-disorder transition temperature T_OD_ = 475 K^2^, only one of these sublattices is fully occupied, while above, Ag^+^ ions are disordered between both with equal probability (*R*-3*m* symmetry), and high ionic conductivity is observed^3^,^4^. Magnetic ordering of the Cr spins is observed below T_N_ = 55 K, and is characterized, according to Rietveld refinement of the neutron diffraction patterns, by the propagation vector **k** = (0 + ε, 0 + ε, 3/2) (ε ~ 0.037(3)), in close agreement with an earlier study^5^. The corresponding magnetic structure can be described as long wavelength antiferromagnetic cycloids running along [110] and stacked antiferromagnetically along *c*, with the Cr spins rotating within the *ab* plane (Fig. 1c). The Cr^+3^ magnetic moment reaches 2.58(2) μ_B_ at 1.5 K, below the expected saturation value of 3 μ_B_, indicating the persistence of a disordered magnetic component at low temperature akin to that observed in other Cr triangular lattices^6^. Low temperature synchrotron diffraction data confirm that there is no lowering of the *R*3*m* symmetry down to 15 K.

AgCrSe_2_ was revisited recently for its thermoelectric properties above room temperature^7^,^8^: above T_OD_, its thermal conductivity is extremely low (~0.2–0.5 W.K^−1^.m^−1^) and nearly temperature independent, a fact related to the amorphous lattice of silver atoms. Figure 2 illustrates the temperature evolution of the thermoelectric power S and thermal conductivity κ below T_OD_, in the 5 to 300 K range. S exhibits a large positive value of 300 μV.K^−1^ at RT, characterizing holes as the majority carriers. It slowly decreases between 300 K and 75 K, with a change of slope being observed below 75 K, without any noticeable change at T_N_. The thermal conductivity of AgCrSe_2_ at RT is extremely low, ~0.4(1) W.K^−1^.m^−1^, a value comparable to that of glasses. Its sluggish increase as T decreases in the range 300–100 K does not follow the T^−1^ behaviour expected for phonon heat transport^9^. A small thermal hysteresis is visible in κ but not in S, suggesting a slight structural irreversibility that has little impact on the electronic states.

The moderately high resistivity of AgCrSe_2_ (~7 mΩ.cm at RT, inset of Fig. 2) and its negative slope with respect to temperature suggest activated electronic transport with a small gap, consistent with the Seebeck coefficient, which is high but not divergent at low temperature^10^. The resistivity vs. T curve does not show any obvious anomaly at T_N_. The inset of Fig. 2 also shows the ρ(T) curve measured in an external magnetic field of 9 T. A positive magnetoresistance is observed up to 150 K. Essentially no field effect is observed in κ or S up to the same magnetic field (Fig. 2).

To investigate in more details the dynamical properties of AgCrSe_2_, inelastic neutron scattering experiments were performed between 10 K and 200 K, as illustrated on Fig. 3. Over the whole temperature range, the main feature of the excitation spectra is clearly a non-dispersive inelastic signal near 3.3 meV (Fig. 3b–f). The increasing intensity of this excitation when temperature increases is characteristic of a bosonic mode. Its existence up to room temperature attests that it is a lattice vibrational mode, while the lack of dispersion shows its localized character. In parallel, the results of the Rietveld refinement of the 300 K synchrotron data (see Supplementary Materials Table I and II) show that the atomic motion of Ag^+^ is anomalously large and strongly anisotropic, confined within the *ab* plane. This feature has also been reported for AgCrS_2_^11^,^12^,^13^, along with the existence of a low frequency mode^14^,^15^, in studies related to the Ag^+^ superionic conductivity at high temperature. To get further information on the dynamics of this system, and particularly on the Ag modes, ab-initio molecular dynamics (AIMD) calculations were performed. The most remarkable feature in the calculated density of states, S(E), is a peak located at 3.3 meV, in very good agreement with the inelastic neutron data, and whose main contribution corresponds to Ag vibrations within the *ab* plane (Supplementary Figure S1). This peak is also remarkably symmetric, suggesting that the modes involving the Ag displacements are localised, i.e., with a flat dispersion in an extended region of the Brillouin zone. This result confirms therefore that the 3 meV mode in AgCrSe_2_ originates from vibrations of the loosely bound silver ions, parallel to the triangular planes. As seen on Fig. 4a, above 70 K, the susceptibility χ”(Q, E) of this mode, which is related to the dynamical structure factor shown in Fig. 3 by the fluctuation-dissipation theorem, is not constant, but starts to decrease with increasing temperature. This is a sign of the enhanced anharmonicity of the motion of the Ag^+^ inside their potential well as temperature increases and nears the order-disorder transition T_OD_. The shape of this effective potential well below T_OD_, as derived from^14^, shows that the largest thermal vibrations are directed towards the octahedral sites, between the CrSe_2_ layers (Fig. 1b), rather than directly towards the empty sites of the β sublattice, which act as virtual cages. Accordingly, AIMD results show that at the temperature of the simulation T = 150 K, some Ag atoms still perform jumps between adjacent positions in the same layer, in contrast with the Cr and Se atoms, whose displacement clouds are localised around equilibrium positions.

Heat conduction is adequately described by a simple phenomenological model, the minimum thermal conductivity, in which a random walk of vibrational energy on the time and length scales of atomic vibrations and interatomic spacings can carry a heat current^16^. Poor thermal conductivity is typically achieved in “phonon glasses”^17^,^18^, in which phonon scattering has been optimized. In electrically insulating amorphous solids, the amorphous structure scatters phonons to mean free paths of the order of the atomic scale, and they exhibit some of the lowest thermal conductivity values. Other concepts of phonon scattering are based on structural disorder: point defects, such as random atomic substitution or vacancies, introduction of heavy atoms as incoherent rattlers in cage-like structures in skutterudites^19^,^20^ or clathrates^21^, or nano-composite microstructures^22^,^23^,^24^, are examples of the strategies used to follow the phonon glass concept. The low value of κ in AgCrSe_2_ indicates that the phonon mean free path in this compound is extremely short, even below the order-disorder transition. Diffraction data confirm the absence of static disorder unambiguously in AgCrSe_2_: the refinement of the occupation level on interstitial tetrahedral (β) or octahedral free sites always leads to a non-significant value, challenging such a scenario. On the other hand, because the heat carrying phonons are the long-wavelength (or low energy) ones, efficient phonon scattering can be expected from the interaction between acoustic phonons and the 3 meV mode characterising the Ag^+^ vibration, and can be considered as a plausible explanation to account for the low thermal conductivity of AgCrSe_2_.

In an insulating magnetic system (the electronic contribution to the thermal conductivity that can be extracted from the transport data according to the Wiedemann-Franz law is actually of the order of 1.1 10^−5 ^W.m^−1^.K^−1^ 10 and can be therefore considered to be negligible), heat is carried primarily by phonons and magnons, and it raises the question as to whether magnetic excitations in AgCrSe_2_ could actually contribute to^25^, or impede through spin-lattice coupling^26^, heat transport properties. This is of particular relevance as the anomalous temperature evolution of the in-plane cell parameters observed in AgCrSe_2_ (Supplementary Figure S2) originates arguably from magneto-elastic coupling, the *ab* plane being the plane in which lay the Cr moments.

In the magnetically ordered phase, a spin-wave dispersive magnetic signal is clearly visible in the excitation spectra: on the 2 K spectrum (Fig. 3a), it stems from the ((000) ± **k)** and ((003) − **k)** Bragg magnetic peaks at Q = 0.5 Å^−1^. The top of the excitation spectrum can be estimated to be ~30 meV, with an intensity maximum around 20 meV. A strongly dispersive magnetic signal, whose intensity peaks below 2 meV, is also observed around Q = 2 Å^−1^ (Fig. 3a–c). To quantitatively analyse the observations, spin wave modelling was carried out, using a simple spin Hamiltonian with nearest (*J*_*ab*_), next-nearest (*J*_*NN*_) neighbour interactions, and coupling between nearest neighbours in adjacent layers (*J*_*C*_). *J*_*ab*_, *J*_*NN*_ and *J*_*C*_ values were carefully chosen to stabilize the experimentally observed propagation vector **k** (ε = 0.037), at the mean field level. The magnetic spectrum of AgCrSe_2_ can be readily modelled within this approach, the best agreement leading to the following exchange parameters: a ferromagnetic *J*_*ab*_ ~ 2.1, and two antiferromagnetic second neighbour *J*_*NN*_ ~ −0.71 and *J*_*C*_ ~ −0.09 meV (Supplementary Figure S3). The *J*_*C*_ parameter value actually directly affects the energy position of the Q = 1.2 Å^−1^ magnetic scattering, so that a weak antiferromagnetic *Jc* is necessary to reproduce correctly the experimental spectrum in this Q range. The fact that *J*_*ab*_ is ferromagnetic in AgCrSe_2_ is theoretically understood considering that for large Cr-Cr distances (~3.69 Å at 2 K), antiferromagnetic direct exchange is overcome by ferromagnetic super-exchange interactions through a nearly 90° Cr-Se-Cr angle^5^,^27^. It agrees with the positive Curie-Weiss temperature θ_CW_ of ~75–80 K, indicative of predominantly ferromagnetic interactions^1^,^10^. The rather large value of *J*_*NN*_ confirms the predominant role of this exchange path, a known characteristic in triangular layer compounds^28^,^29^.

The dispersive magnetic signal observed around Q = 2 Å^−1^ on Fig. 3a actually persists in the magnetically disordered phase, far above T_N_, up to 200 K, as illustrated on Fig. 3b–f. Above T_N_, there is no theoretical model to describe high energy spin fluctuations, as the lack of long-range magnetic ordering (inferred from the absence of magnetic Bragg peaks in the elastic scattering experiments) precludes the spin wave formalism. The fairly low value of *J*_*C*_ is nevertheless indicative of weak inter triangular planes coupling, and the persistence of a magnetic signal above T_N_ is ascribed to dispersive two-dimensional collective excitations within the *ab* plane^30^,^31^,^32^. A decrease in the steepness of the signal (arrow on Fig. 4b), as well as a broadening of the Q width, indicate a decrease of the in-plane exchange by ~30% between 10 K and 150 K.

The temperature range of existence of these 2D excitations seems to be correlated with the negative thermal expansion of AgCrSe_2_ in the *ab* plane seen below 150 K (Supplementary Figure S2). However, even if a substantial magneto-elastic coupling is at play, the impact of the magnetic fluctuations on κ remains difficult to ascertain. Indeed, upon cooling there is a sharp increase of κ below the onset of antiferromagnetic order at T_N_ (Fig. 2b), which could be interpreted as owing to the freezing of the magnetic fluctuations upon entering the ordered state. The lack of any field effect on κ prevents however any further identification of the impact of the magnetic fluctuations or excitations on thermal transport, as it either means that the applied magnetic field is not large enough, or that the actual phonon scattering described above dwarfs any other effects.

The main originality of the localised mode in AgCrSe_2_ is that it derives from the trapping of Ag^+^ in a potential rather than in a physical cage, bearing resemblance to the low energy modes involving the motion of heavy side groups in some crystalline polymers^33^, or to the rattling of Na^+^ in three-vacancy clusters^34^. This low energy mode actually appears to be an intrinsic feature of the tetrahedral sites in this structural type, as it has been reported for other isostructural compounds to AgCrSe_2_, like CuCrS_2_^35^ or AgCrS_2_^15^ (Supplementary Figure S4). This provides an interesting mean to further decrease the thermal conductivity in this substructured system, by replacing Ag^+^ with heavier atoms, like Au^+^, in the phonon-glass layer, or by changing the ligand ion to alter chemical bonding and modify the potential well. At this stage, further first-principle studies would be necessary to apprehend the acoustic phonon scattering mechanism induced by an array of anharmonic potentials, and how it compares with the mechanism based on a wideband three-phonon scattering process proposed for rattlers in clathrates, which leads to a severe reduction of the acoustic phonon average relaxation time, rather than of their velocity^36^,^37^. The next step would then be to exploit the additional degree of freedom provided by the spin fluctuations to control heat transport properties. Without any knowledge on the nature of the coupling between phonons and spin fluctuations in AgCrSe_2_, this remains fairly prospective, but an idea would be to enhance magneto-elastic coupling in the transition metal layer to induce phonon scattering by spin fluctuations. The substructure rationale imposes only that any inter-layer coupling should be carefully controlled so as to preserve each layer’s intrinsic properties.

## Methods Summary

5g of polycrystalline AgCrSe_2_ were prepared by high temperature solid state reaction. Powders of Ag, Cr and Se precursors were weighted according to the stoichiometric ratio. The resulting powder was carefully ground, pressed in the shape of bars, and heated in an evacuated silica tube at 900 °C for 24 hrs. The sample was then checked by room temperature X-ray diffraction and found to be single phase.

Synchrotron X-ray diffraction was performed on the CRISTAL beamline (Soleil Synchrotron, Saint-Aubin, France) and on the MSPD beamline (ALBA Synchrotron, Barcelona, Spain). The powder sample was put in a glass capillary tube of 0.3 mm inner diameter, and rotated during the experiment. The data was collected at 300 and 15 K, using a wavelength *λ* = 0.45678 Å at Soleil, and at 300 and 80 K using a wavelength λ = 0.62020 Å at ALBA.

**Figure 3:**
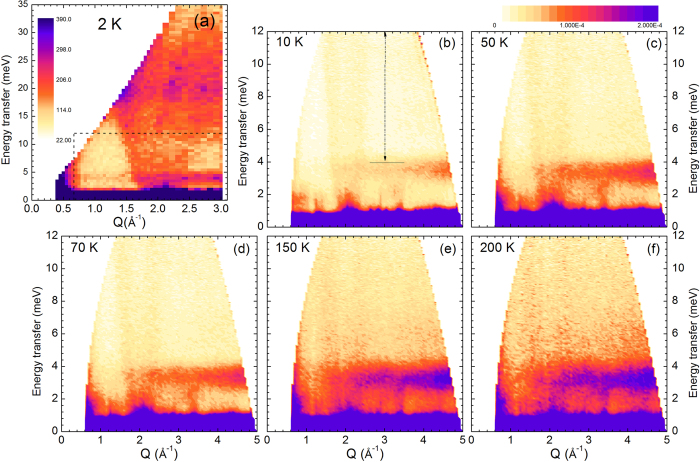
Temperature evolution of the dynamical properties of AgCrSe_2_, showing the localized lattice mode at 3 meV and the dispersive magnetic signal at Q = 2 Å^−1^. Figure 3a is an overview of the spectrum up to 35 meV (triple-axis, *k*_*f*_ = 2.662 Å^−1^) at 2 K, Fig. 3b–f (time-of-flight data, λ_*i*_ = 2.2 Å) are a higher resolution mapping of the E < 12 meV area (corresponding to the dashed zone of Fig. 3a) at different temperatures.

Neutron powder diffraction (NPD) versus temperature was performed on the G4.1 diffractometer (LLB-Orphée, CEA-Saclay, France) from 1.5 to 300 K, using a wavelength λ = 2.425 Å. Rietveld refinements were performed with programs of the FullProf suite^38^. Inelastic neutron scattering experiments were performed on the thermal (2T, *k*_*f*_ = 2.662 Å^−1^) neutron triple-axis spectrometers at LLB-Orphée. The collimation used was 60’, leading to an energy resolution of ~1 meV. Higher order contaminations were removed using two pyrolytic graphite filters placed in the scattered beam; the use of two filters was necessary to ensure complete removal of higher order contamination in the 20 meV zone, of particular importance to study the magnetic spectrum of AgCrSe_2_. Time-of-flight (TOF) inelastic neutron scattering experiments were performed using the thermal spectrometer IN4 at the Institut Laue-Langevin (ILL, Grenoble), with various incident wavelength settings (λ_i_ = 1.1 Å, 2.2 Å and 3.0 Å), between 10 K and 150 K^39^. At 2.2 Å, the instrumental resolution in energy is ~0.5 meV. The colour maps which are shown on Fig. 3b–f have been obtained by normalising the data by the detector efficiency before removing the background signal of an empty cell. The detector calibration file was obtained by measuring a flat vanadium sample at the appropriate wavelength (2.2 Å in this case). The constant Q cuts were obtained by integrating the data over dQ = 0.05 Å.

To model the spin dynamics, spin-wave calculations were performed using the Spinwave software developed at LLB^40^, and recently extended to treat incommensurate magnetic structures with any single propagation vector. Based on the Holstein-Primakov approximation, the code diagonalizes the chosen spin Hamiltonian; in the present case, the calculations were performed using isotropic exchange couplings and an easy-plane anisotropy term perpendicular to [001]. S(Q, ω) is first calculated integrating over a sphere in the reciprocal space, sampling the sphere following a Fibonacci based algorithm^41^.

Resistivity, thermal conductivity and thermoelectric power measurements were performed via a standard 4 terminal technique, using the Thermal Transport Option of the Physical Properties Measurement System (PPMS) from Quantum Design. The values of κ obtained above room temperature in^8^ are based on the measurement of thermal diffusivity using the laser flash technique, which require additional measurement of the specific heat to extract κ. The values of κ presented in this article are from a direct steady-state measurement, which do not involve the specific heat. They are very accurate at low temperature, but can suffer from errors due to radiation loss at room temperature, so that we estimate an uncertainty of approximately ±20% on the room temperature value of κ. Magnetisation measurements were carried out using a SQUID (MPMS, Quantum Design) magnetometer.

Molecular dynamics (MD) calculations were performed using the Density Functional Theory (DFT) code VASP^42^,^43^,^44^, under the Generalized Gradient Approximation (GGA) of the exchange-correlation functional, as formulated by Perdew *et al*.^45^. Pseudo augmented wave pseudopotentials (paw) were used^46^. The Cr_pv paw was chosen during the complete set of simulations, and an energy cut off equal to 268 eV was used. The simulation box is a quadratic supercell built from 18 hexagonal unit cells. It contains 216 atoms (54 Ag, 108 Se, 54 Cr). The DFT electronic loops were calculated at the Γ point only. The atomic partial density of states were calculated from the Fourier Transform of the velocity autocorrelation function, using nMoldyn v3.0^47^.

## Additional Information

**How to cite this article**: Damay, F. *et al*. Localised Ag^+^ vibrations at the origin of ultralow thermal conductivity in layered thermoelectric AgCrSe_2_. *Sci. Rep.* **6**, 23415; doi: 10.1038/srep23415 (2016).

## Supplementary Material

Supplementary Information

## Figures and Tables

**Figure 1:**
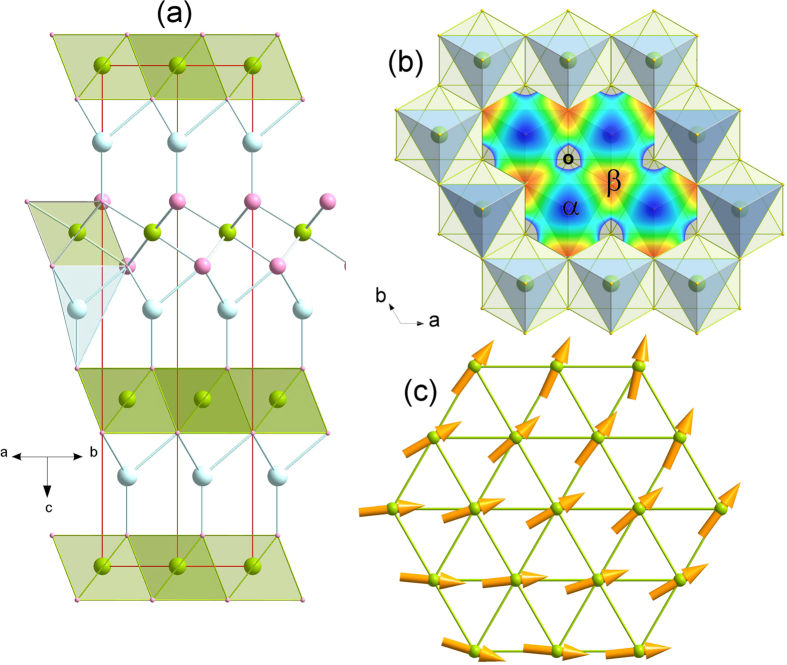
Crystal and magnetic structures of AgCrSe_2_. (**a**) *R*3*m* crystal structure of AgCrSe_2_. Ag, Cr and Se atoms are drawn in light blue, green and pink, respectively. Coordination polyhedra, octahedral for Cr and tetrahedral for Ag, are also shown. (**b**) illustrates the compact CrSe_2_ layer on which is superimposed the energy map of the effective potential surrounding Ag^+^ in its tetrahedron, following^14^. O indicates octahedral (empty) sites. α and β label the two Ag^+^ triangular sublattices, full and empty, respectively in the *R*3*m* structure. The long-wavelength cycloidal magnetic ordering below T_N_ = 55 K is shown in (**c**) in the *ab* plane, the coupling between planes being antiferromagnetic.

**Figure 2:**
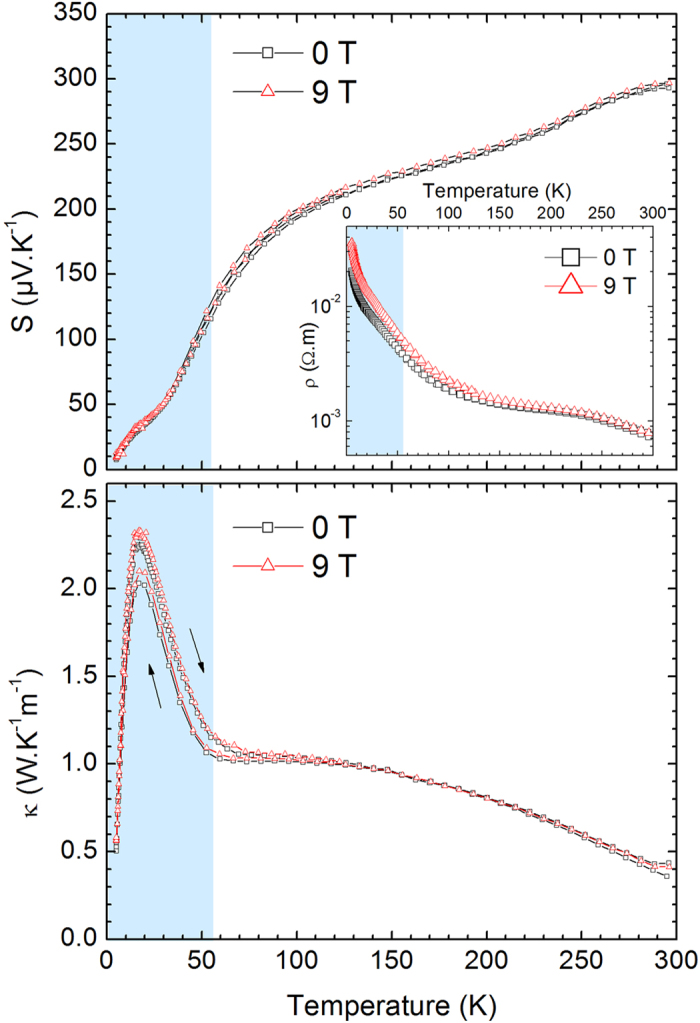
Thermoelectric power, thermal and electrical conductivity of AgCrSe_2_. Thermoelectric power S (top), thermal conductivity κ (bottom), and electrical resistivity (inset) vs. temperature are measured in 0 (square symbols) and 9 T (triangular symbols).

**Figure 4:**
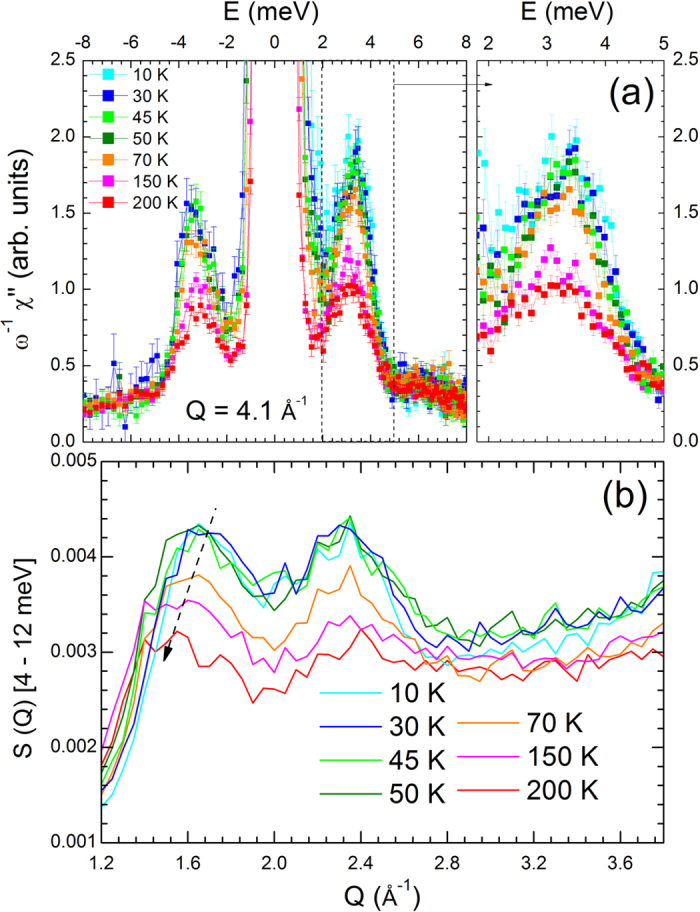
Increasing anharmonicity of the lattice vibration and persisting magnetic excitations in AgCrSe_2_ for T ≫ T_N_, exemplified through the evolution with temperature of (**a**) constant Q = 4.1 Å^−1^ cuts (symmetric in energy with respect to the elastic line) and (**b**) S(Q) integrated between 4 and 12 meV (as shown by the vertical arrow on Fig. 3b). All these cuts were extracted from the time-of-flight neutron data at λ_*i*_ = 2.2 Å, see the Experimental section for more details. The arrow on Fig. 4b illustrates the shift of the intensity maximum towards smaller Q as temperature increases, a sign of the decrease of the steepness of the dispersion.
