# Supplementary material for: Localised Ag+ vibrations at the origin of ultralow thermal conductivity in layered thermoelectric AgCrSe2
Source: Sci Rep. 2016 Mar 22;6:23415. doi: 10.1038/srep23415 (PMC4802330; doi:10.1038/srep23415)
Supplement: Supplementary Information [file srep23415-s1.pdf]

Localised Ag^+ vibrations at the origin of ultralow thermal conductivity in layered thermoelectric $AgCrSe_2$

F. Damay[1], S. Petit[1], S. Rols[2], M. Braendlein[1], R. Daou[3], E. Elkaïm[4], F. Fauth[5], F. Gascoin[3], C. Martin[3] and A. Maignan[3]

Supplementary material

Table S1: Rietveld refinement results of high resolution synchrotron X-ray powder diffractogram of $AgCrSe_2$ at 295 K and 80 K (space group *R3m* (n°160, H setting) with all atoms on Wyckoff position 3*a* (0, 0, *z*)).

Temperature	295 K	80 K
Cell parameters (Å)		
a	3.6836(2)	3.6844(2)
c	21.2375(12)	21.11561(10)
Cell volume *V* (Å^3)	249.6(2)	248.2(2)
Ag (0, 0, *z*)	0.1529(3)	0.1513(2)
Cr (0, 0, *z*)	0	0
Se_1 (0, 0, *z*)	0.2713(3)	0.2709(3)
Se_2 (0, 0, *z*)	0.7337(3)	0.7336(3)
$U_{anisotropic}$ (Å^2)		
Ag U_{11}	0.081(6)	0.016(7)
Ag U_{33}	0.004(2)	0.010(3)
Nb. of reflections	63	63
Nb. of parameters	17	17
Bragg R factor	3.88	4.28
χ^2	2.04	3.18

Table S2: Selected inter-atomic distances and angles in $AgCrSe_2$ at 295 K and 80 K (from high resolution synchrotron X-ray powder diffractograms).

Temperature	295 K	80 K
Distances (Å)		
Ag-Se_1	2.5162(1) (x1)	2.5284(1)
Ag-Se_2	2.8025(4) (x3)	2.7792(4)
Cr-Se_1	2.4970(5) (x3)	2.5005(3)
Cr-Se_2	2.5624(5) (x3)	2.5505(5)
Cr-Cr_{in}	3.6836(2) (x6)	3.6844(1)
Cr-Cr_{out}	7.3918(4) (x6)	7.3530(4)
Cr-Cr_{in} (2nd neighbour)	6.380(1) (x6)	6.381(1)
$CrSe_2$ layer thickness (Å)	2.738(1)	2.721(1)
$AgSe_4$ height (Å)	4.341(1)	4.317(1)
Angles (°)		
Se-Cr-Se_{in}	91.91(3)	92.49(3)
Se-Cr-Se_{out}	86.49(3)	86.29(3)
S-Ag-S	130.63(3)	130.06(3)

Figure S1 : (Left) Total (black) and partial (red : Ag, blue : Cr, magenta : Se) simulated structure factors obtained from the ab-initio molecular dynamics calculations at 150 K. (Right) Partial density of state for the Ag atoms, projected along the [110] axis (« in plane » vibrations, in blue), and along the [001] direction (« out of plane » vibrations, in red).

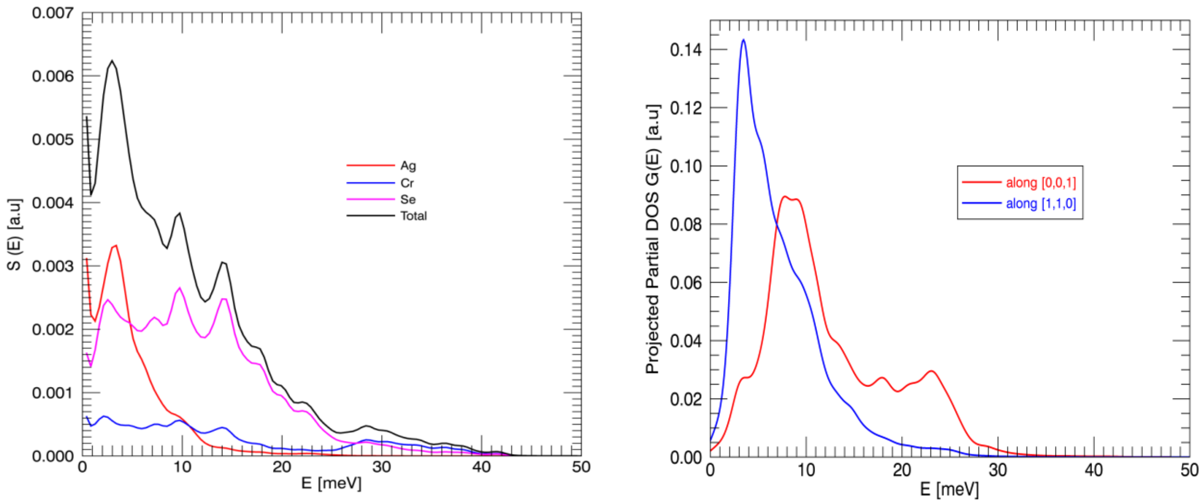

Figure S2 : Temperature evolution of the $AgCrSe_2$ cell parameter *a* and cell volume *V*. The red line is a Debye fit, and the blue line is a guide to the eye.

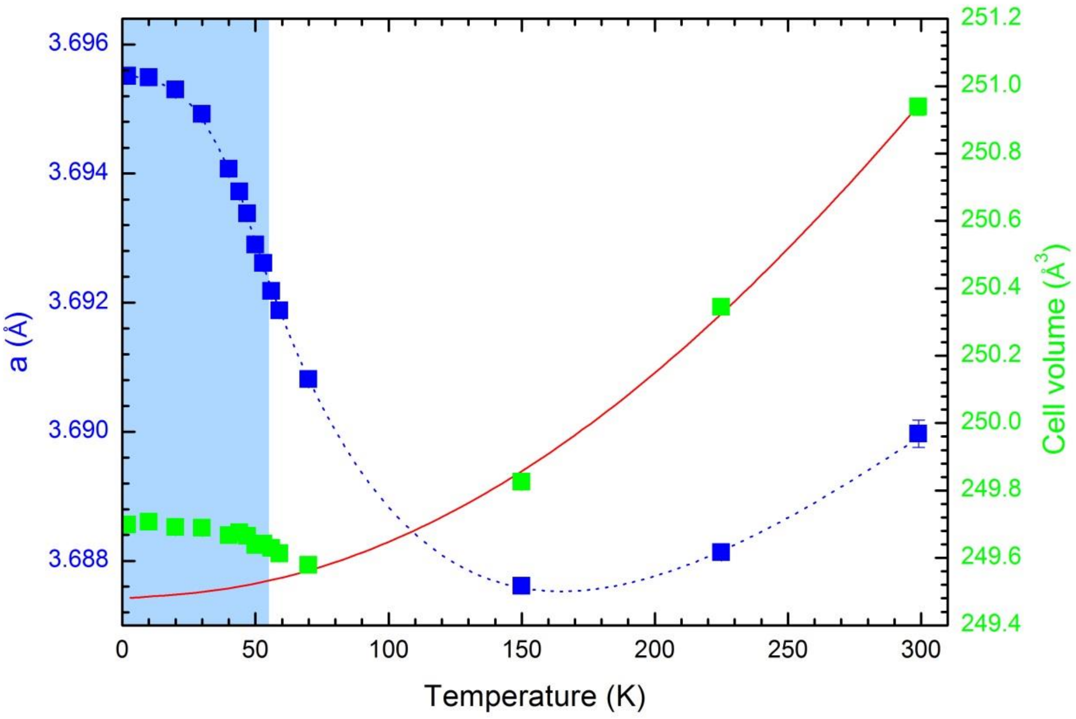

Figure S3 : Spin-wave calculation performed modelling the incommensurate magnetic structure of $AgCrSe_2$ at 2 K, with the following magnetic exchange values: J_{ab} ~ 2.1 meV, J_{NN} ~ -0.71 meV and J_C ~ -0.09 meV (see text for definition of J's).

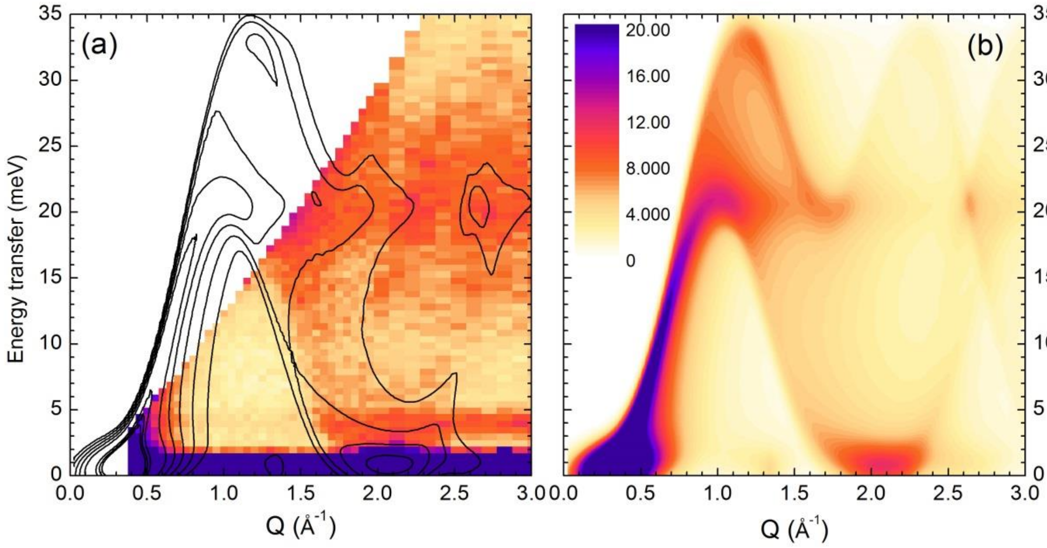

Figure S4 : Dynamical properties of $AgCrS_2$. The red arrow indicates the localised mode attributed to the in-plane vibration of Ag^+.

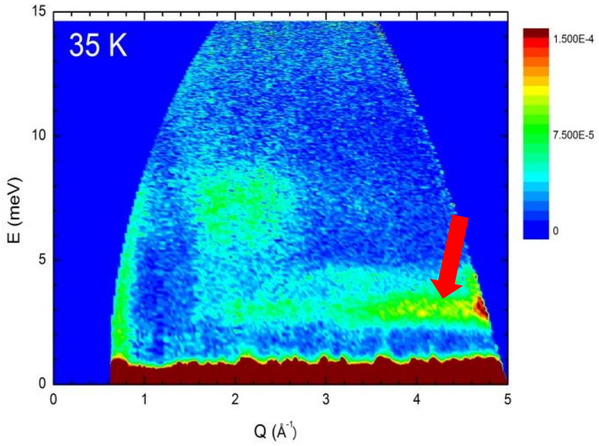

$AgCrS_2$
